# Transient Inactivation of Shell Part of Nucleus Accumbens Inhibits and Exacerbates Stress-Induced Metabolic Alterations in Wistar Rats

**DOI:** 10.18869/nirp.bcn.8.2.121

**Published:** 2017

**Authors:** Mina Ranjbaran, Hassan Aghaei, Vahdat Hajihoseinlou, Hedayat Sahraei, Katayoon Ranjbaran

**Affiliations:** 1. Neuroscience Research Center, Baqiyatallah University of Medical Sciences, Tehran, Iran.; 2. Department of Biology, Faculty of Science, Campus of Shahid Bahonar, Farhangiaan University, Hamadan, Iran.

**Keywords:** Stress, Lidocaine, Nucleus accumbens, Corticosterone, Locomotor activity, Rearing

## Abstract

**Introduction:**

The role of different parts of the extended amygdala in metabolic signs of stress is not well understood. In the present study, we decided to evaluate the impact of the shell part of nucleus accumbens (NAc) on metabolic disturbance induced by electro foot shock stress using transient inactivation method in the rat.

**Methods:**

Male Wistar rats (W: 230–250 g) were canuulated unilaterally in the shell part of nucleus accumbens and left one week for recovery. Five minutes before each stress session, the animals either received sterile saline (0.25 μl/side) (control) or lidocaine 2% (0.25 μl/side) (experiment). Blood samples were taken from rats’ retro-orbital sinus for plasma corticosterone measurements. In addition, animals’ weight gain, food and water intake, locomotor activity, and rearing were recorded.

**Results:**

Stress reduced weight gain and food intake, increased water intake and plasma corticosterone level, and reduces locomotor activity and rearing. Transient inactivation of the right side of the NAc inhibits the stress effect on weight gain, water intake and plasma corticosterone level, but not food intake. However, when the left side of the NAc was inactivated, only weight gain was affected and other parameters were not differing from stress group. Even thought, the plasma corticosterone level was elevated.

**Conclusion:**

In conclusion, our data indicated that right side of shell part of NAc transient inactivation leads to reduction in metabolic signs of stress but left side of shell part of the NAc inactivation even exacerbates stress signs.

## 1. Introduction

Stress exposure can induce several side effects, including behavioral and metabolic disturbances (Smith & Vale, 2006), which are thought to be mediated by the alteration of brain function under stress conditions (Kovács, 2013; Picard et al., 2014; McEwen, 2012). However, the mechanisms by which stress influences the activities of living organisms are not fully understood nor are identities of the brain areas in which these mechanisms are mediated (McEwen, 2012; Hunter and McEwen, 2013). Studies have revealed that hormones and neurotransmitters released during stressful events can mediate stress effects (McEwen, 2012). The most important activity identified in the brain and its periphery after stress exposure is arguably the change in cell morphology and function that takes place in the primary parts of the brain and body responsible for stress, e.g., the adrenal medulla, amygdala, hippocampus, and prefrontal cortex (McEwen and Morrison, 2013; Roozendaal et al., 2009; Roozendaal et al., 1996; Belujon and Grace, 2011; Pape and Pape, 2010). However, little attention has been paid to the role of other parts of the brain, such as the nucleus accumbens (NAc), in the mediation or regulation of stress effects and/or responses (Schwienbachera et al., 2004; Rothwell et al., 2011). The shell of the NAc and the central amygdala form part of the functional and anatomical compartment in the brain known as the extended amygdala (Koob, 1999).

The extended amygdala, particularly the central nucleus of the amygdala, is involved in the response to acute and chronic stress (Koob, 1999; Roozendaal et al., 1996). The NAc shell acts in concert with the central amygdala to ameliorate the emotional responses to various stress states, including fear and addiction (Koob, 1999). The shell of the NAc consists mainly of neural fibers with only a scant number of neurons, which are mainly gamma-aminobutyric acid (GABA)-ergic medium spiny neurons (Jongen-Rêlo et al., 1994). As its main input, the NAc shell receives dopaminergic axons from dopaminergic neurons in the ventral tegmental area (Voorn et al., 1986). Although a number of comprehensive studies have been published regarding the role of the NAc shell in brain functions such as drug addiction (Koob, 1999) and feeding behavior (Bello and Hajnal, 2010), its role in other functions such as the response to stress remain to be explored. In addition, the hormones and reactive oxygen species released during stress affect the body (and cell) metabolism (Picard et al., 2014; Dallman et al., 2003). In the present study, we attempted to address the effects of transient inactivation of the left or right side of the NAc shell region on the stress-induced disturbance of metabolic and behavioral responses in rats.

## 2. Methods

### 2.1. Animals

Male Wistar rats (250±20 g, purchased from Pasture Institute, Tehran, Iran) were used in this study (n=8 rats/ group). The animals were initially kept in groups of four per cage with a 12:12 h lightcycle (lights on at 07.00 pm) and ad libitum access to food and water. Prior to the experiment, they were randomly allocated to an experimental group. The experiments were conducted in accordance with the ethical guidelines and approved by the local ethics committee (the Baqiyatallah University of Medical Sciences Committee on the Use and Care of Animals).

### 2.2. Drugs

The following drugs were used in the study: Lidocaine hydrochloride (Sigma, USA), ketamine hydrochloride (Alfasan Worden, Holland), and diazepam hydrochloride (Rooz Darou, Iran). The drugs were dissolved in sterile saline. Lidocaine 2% was prepared and administered intra-NAc at 5 min before each stress session at a volume of 1 μl/rat. The control groups received sterile saline.

### 2.3. Apparatus

The apparatus used for stress induction consisted of Plexiglas supplied by the Borj-e-Sanat corporation, Tehran, Iran (Hooshmandi et al., 2011) and nine equal compartments (16×16×54 cm). The apparatus floor was equipped with stainless steel rods (4 mm in diameter), which were placed 1.3 cm apart. The rods were attached to a generator that was controlled by a computer. The generator produced an electrical current of 0.1 mA, which was applied to generate a 100-s electric shock to the rats’ feet.

### 2.4. Experimental design

Electric foot shock stress was applied for seven consecutive days between 09:00 and 16:00. Animals were transferred to the experimental room 1 h before the experiments began for environmental adaptation. Subsequently, the animals in the stress group were placed individually in the aforementioned compartments; after 30 min, an electric foot shock was administered (0.1 mA for 100 s). After stress termination, the animals remained in the compartment for an additional 30 min before being returned to their home cages. The animals in the control group were placed in the compartments for 60 min without being subjected to foot shock. The time for stress induction for each animal was randomly selected for minimum stress adaptation.

### 2.5. Animal grouping

The animals were divided into six groups (n=8/group). Control group received no stress and did not undergo surgical procedure. Stress group received stress but did not undergo surgical procedure. CNTL-R and CNTL-L groups received stress and undergo surgical procedure but injected with saline in right (CNTL-R) or left (CNTLL) part of nucleus accumbens shell region as positive control groups for lidocaine. EXP-R and EXP-L groups received stress and surgical procedure and injected with lidocaine into their nucleus accumbens shell region.

### 2.6. Metabolic parameters recording

The weight of the animals was recorded on days 1 and 7 before the stress session. In addition, the amount of food and water consumed by each animal on these days was recorded as a measure of other metabolic factors of stress.

### 2.7. Animals’ behaviour recording

The behavior of each animal was digitally videoed for 10 min. Video files were later analyzed offline by a person who was not familiar with the experiments. Dopa-mine-related behaviors, including locomotion activity and rearing, were distinguished during analyses.

### 2.8. Blood sampling

Blood samples were collected from the rats’ retro-orbital sinus (0.9 ml of blood in 0.1 ml of 1% sodium citrate). The samples were centrifuged at 3000 rpm and 4°C for 5 min, and the supernatant fluid was collected for use in a corticosterone assay. The corticosterone was determined using an ELISA kit (Rat Corticosterone ELISA kit; EIA-4164; DRG Instruments GmbH, Germany) at 450 nm.

### 2.9. Surgical procedures

The rats were anesthetized with ketamine hydrochloride (70 mg/kg, i.p.) + xylazine (10 mg/kg, i.p.) and one 23-gauge stainless steel cannula was placed stereotaxically (Stolting Instruments, USA) into the shell of the NAc at a position 500 μm above the intended site of injection according to the atlas of Paxinos and Watson (1987). The stereotaxic coordinates for the NAc shell were as follows: Incisor bar (−3.3 mm), 1.2 mm anterior to the bregma, ±0.8 mm lateral to the sagittal suture, and 6.8 mm down from the top of the skull. The cannula was secured to jewelers’ screws with dental acrylic. After completing the surgery, a dummy inner cannula was inserted into the guide cannula and left in place until the injections were made. The length of the dummy cannula was identical to that of the guide cannula. Animals were allowed 7 days to recover from the surgery and anesthesia. For drug infusion, the animals were restrained by hand; the stylets were removed from the guide cannulas and replaced with 30-gauge injection needles (500 μm below the tip of the guide cannula). The solutions were slowly administered at a total volume of 0.25 μl/rat over a period of 60 s. Injection needles were left in place for an additional 60 s to facilitate the diffusion of the drugs.

### 2.10. Histology

**Figure 1.**
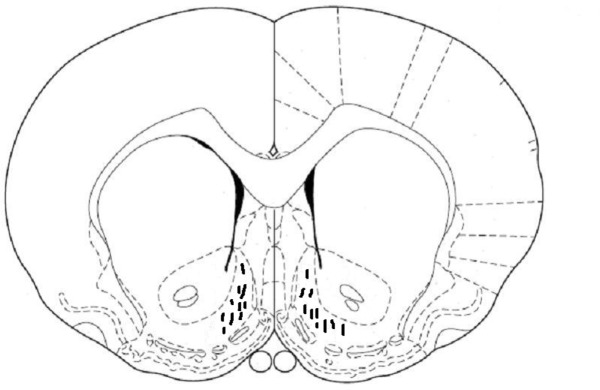
The location of cannula tips in the shell of the nucleus accumbens of animals used in this study. Symbols (|) indicated the position of the cannula tips.

After the completion of testing, all animals were anesthetized and received a transcardiac perfusion with 0.9% normal saline followed by 10% buffered formalin. The rats’ brains were removed, blocked, and cut coronally into 40-μm sections through the cannula placements. The brain tissues were stained with cresyl violet and examined using light microscopy by an observer who was unfamiliar with the behavioral data. Only the animals with correct cannula placements were included in the data analysis (Figure. 1).

### 2.11. Data analysis

All data are expressed as mean±S.E.M. Three-way analysis of variance (ANOVA) followed by Tukey post hoc was applied to evaluate the differences between the groups using side, stress and lidocaine as factors. Differences with P<0.05 were considered significant.

## 3. Results

### 3.1. Effects of lidocaine intra-NAc administration on weight gain with or without stress

**Figure 2.**
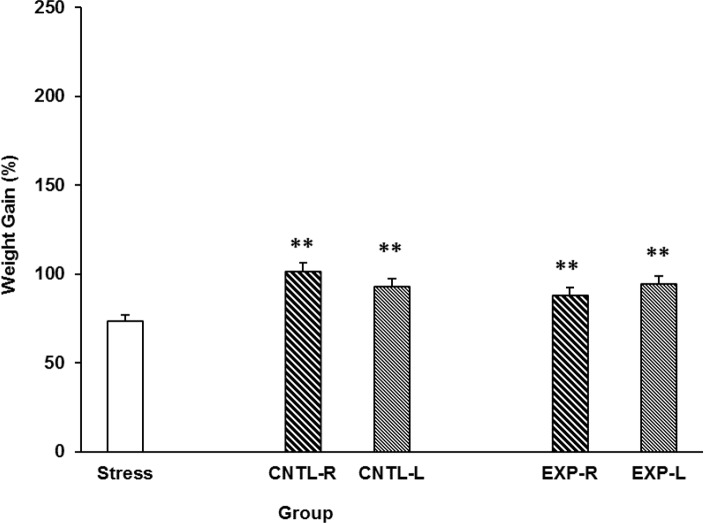
Effects of left or right side transient inhibition of the nucleus accumbens shell on animals’ weight gain under chronic electroshock stress. Each point shows the mean±S.E.M. of weight gain for eight rats; **P<0.01 compared with the stress group. CNTL-R=Control-Right, CNTL-L=Control-Left, EXP-R=Experiment-Right, and EXPL=Experiment-Left.

Stress effect on animals weight gain is shown in Figure 2. Stress decreased animals’ weight gain and transient inactivation of both left and right side of the NAc inhibit the stress effect. Interestingly, saline administrations in the NAc also inhibit the stress effect. [Three-way ANOVA within-group comparison: Side effect: F(3, 18)=0.12, P>0.05, Stress effect: F(4, 24)=2.761, P<0.01, Lidocaine effect: F(1, 16)=1.006, P>0.05, Side×Stress×Lidocaine effect: F(5, 32)=1.854, P<0.05]. Further analysis indicate that stress have a significant effect on animals weight gain (Figure 2).

### 3.2. Intra-NAc transient inhibition effects on stress-reduced food intake

**Figure 3.**
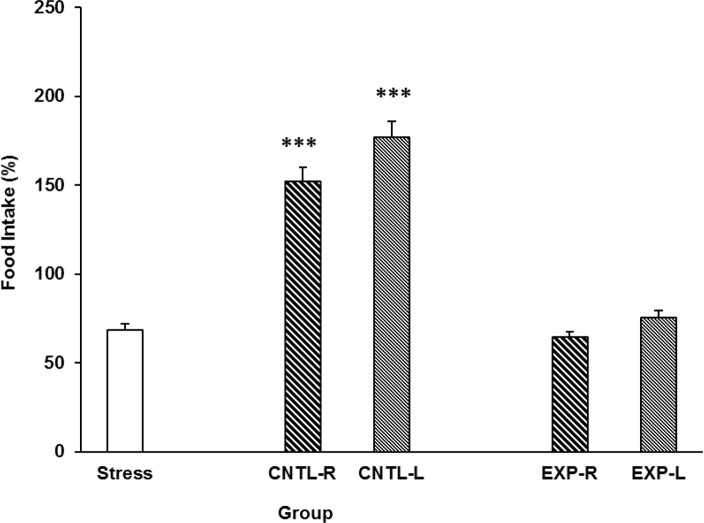
Effects of transient inhibition of the left or right side of the nucleus accumbens shell on animals’ food intake under chronic stress. Each point shows the mean±S.E.M. of food intake for eight rats; ***P<0.001 compared with the stress group. CNTL-R=Control-Right, CNTL-L=Control-Left, EXPR=Experiment-Right, and EXP-L=Experiment-Left.

Data indicated that stress group consume less food in comparison to the control group (Figure 3). However, transient inactivation of the NAc shell part did not inhibit the stress effect on the animals’ food intake. Moreover, animals food consumption increased when the NAc was injected with saline [Three-way ANOVA within-group comparison: Side effect: F(3, 18)=3.81, P<0.001, Stress effect: F(4, 24)=3.502, P<0.01, Lidocaine effect: F(1.16)=2.68, P<0.01, Side×Stress×Lidocaine effect: F(5, 32)=5.342, P<0.001] (Figure 3).

### 3.3. Effects of transient inactivation of NAc on stress-induced water intake

**Figure 4.**
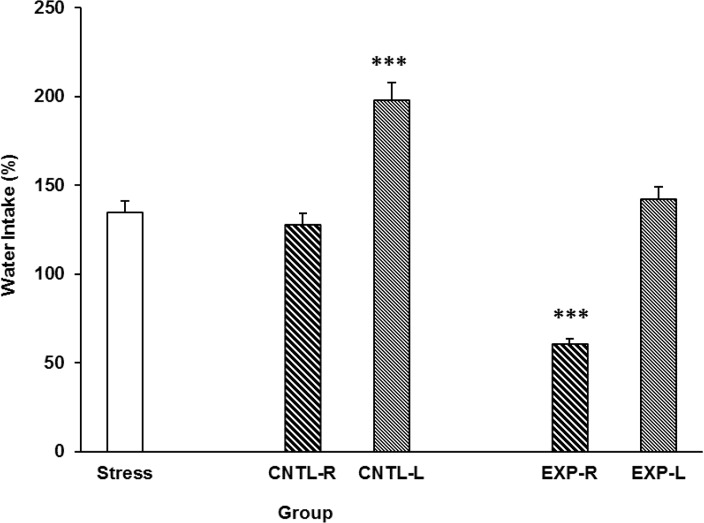
Effects of transient inactivation of the left or right side of the nucleus accumbens shell on animals’ water intake under chronic stress. Each point shows the mean±S.E.M. of water intake for eight rats; **P<0.01 compared with the stress group. CNTL-R=Control-Right, CNTL-L=Control-Left, EXPR=Experiment-Right, and EXP-L=Experiment-Left.

The amount of rats’ water intake after stress is shown in Figure 4. As it is clear in the Figure 4, stress induces water intake and right but not left NAc shell part inhibition reduces the stress effect. However, saline exacerbates stress effect when injected into the NAc shell but the elft side is more prefunded in this regard (Figure 4). [Three-way ANOVA within-group comparison: Side effect: F(3, 18)=4.67, P<0.001, Stress effect: F(4, 24)=3.2, P<0.01, Lidocaine effect: F(1, 16)=5.3, P<0.001, Side×Stress×Lidocaine effect: F(5, 32)=6.32, P<0.001].

### 3.4. Evaluation of transient NAc inactivation on stress-induced plasma corticosterone elevation

**Figure 5.**
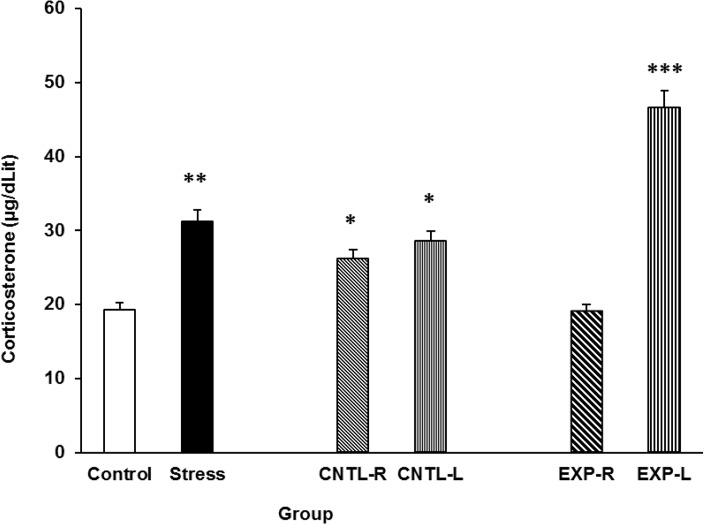
Plasma corticosterone concentration elevation under chronic stress following transient inactivation of the left or right side of the nucleus accumbens shell. Each point shows the mean±S.E.M. of plasma corticosterone for eight rats; ***P<0.001, **P<0.01, and *P<0.05 compared with the control group. CNTLR=Control-Right, CNTL-L=Control-Left, EXP-R=Experiment-Right, and EXP-L=Experiment-Left.

The effects of stress on the rats’ plasma corticosterone concentrations and the effectiveness of transient NAc shell part inhibition on this effect are presented in the Figure 5. Stress increased plasma corticosterone levels and right NAc shell part inhibition reduces the stress effect. Interestingly, left NAc shell part inhibition exacerbates the stress effect on plasma corticosterone level elevation. [Three-Way ANOVA within-group comparison: Side effect: F(3, 18)=6.1, P<0.001, Stress effect: F(4, 24)=4.23, P<0.001, Lidocaine effect: F(1, 16)=4.78, P<0.001, Side×Stress×Lidocaine effect: F(5, 32)=5.72, P<0.001].

### 3.5. Effects of intra-NAc lidocaine administration on stress-induced alteration in locomotion

**Figure 6.**
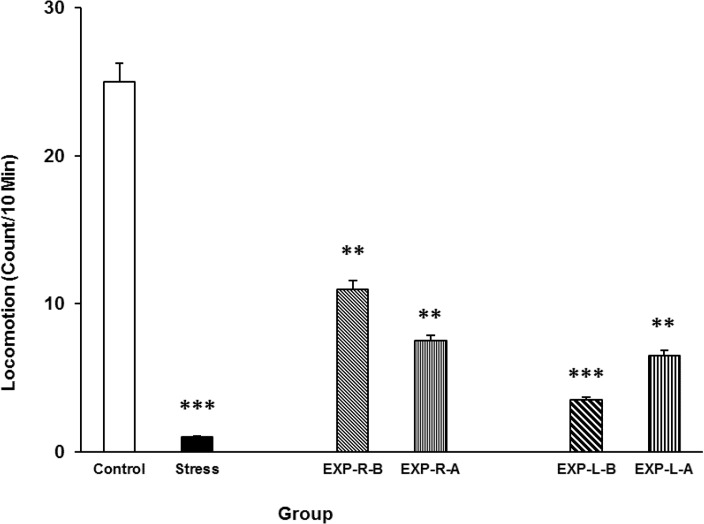
Locomotor activity under chronic stress following transient inactivation of the left or right side of the nucleus accumbens shell. Each point shows the mean±S.E.M. of locomotor activity for eight rats; ***P<0.001 and **P<0.01 compared with the control group. CNTL=Control, EXP-R-B=Experiment-Right-Before, EXP-R-A=Experiment-Right-After, EXP-L-B=Experiment-Left-Before, and EXP-L-A=Experiment-Left-After.

As shown in Figure 6, rats’ locomotion reduced in the stress group. Intra-NAc shell part injection of lidocaine reduces stress effect not completely. Interestingly, it seems that the right side inhibition is more effective in this regard than the left side. [Three-way ANOVA within-group comparison: Side effect: F(3, 18)=3.09, P<0.01, Stress effect: F(4, 24)=4.72, P<0.001, Lidocaine effect: F(1, 16)=2.177, P<0.01, Side×Stress×Lidocaine effect: F(5, 32)=4.33, P<0.001] (Figure 6).

### 3.6. Effects of intra-NAc lidocaine administration on stress-induced alteration in rearing

**Figure 7.**
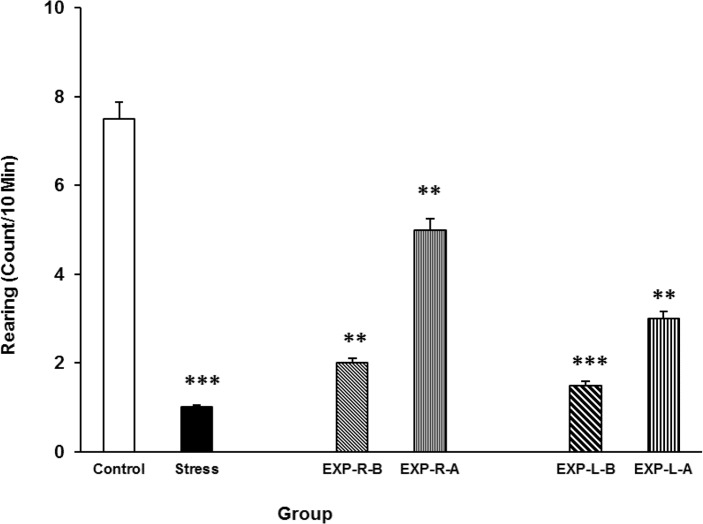
Rearing behavior under chronic stress following transient inactivation of the left or right side of the nucleus accumbens shell. Each point shows the mean±S.E.M. of rearing for eight rats; ***P<0.001 and **P<0.01 compared with the control group. CNTL=Control, EXP-R-B=Experiment-Right-Before, EXP-R-A=Experiment-Right-After, EXP-L-B=Experiment-Left-Before, and EXP-L-A=Experiment-Left-After.

Stress also reduced the number of rearing behaviour in the rats as compared with control group. Again, intra-NAc shell part lidocaine administrations ameliorate the stress effect as well (Figure 7). Further analysis indicated that the right side inhibition has more effect on stress than the left side. [Three-way ANOVA within-group comparison: Side effect: F(3, 18)=3.41, P<0.001, Stress effect: F(4, 24)=4.02, P<0.01, Lidocaine effect: F(1, 16)=3.21, P<0.01, Side×Stress×Lidocaine effect: F(5, 32)=5.23, P<0.001].

## 4. Discussion

The results presented in this research indicated that chronic electro foot shock stress applied in a random pattern produced a broad spectrum of metabolic disturbances in the male rats. Moreover, transient inactivation of the NAc shell interacted with the stress effects in a side-biased manner. According to these findings, one can conclude that the NAc shell may plays an important role in animal’s responses to stress.

These data showed that stress increased plasma corticosterone levels in male rats. Several findings from studies that used animal models, including both rats (Ghalami et al., 2013; Hooshmandi et al., 2011) and mice (Halataei et al., 2011), support these results and indicate that electro foot shock stress induces plasma corticosterone elevation in these animals. It is now clear that stressful events can activate parvocellular neurons within the paraventricular nucleus in the hypothalamus (Popoli et al., 2011), and these neurons secrete their corticotropin releasing factor (CRF) content in the hypothalamus-pituitary portal vein blood stream (Smith and Vale, 2006; Kovács 2013; Guillaume et al., 1987). Indeed, CRF activates the pituicytes for the release of adreno-corticotropin hormone, which can induce the production of adrenal glucocorticoids, such as corticosterone in the cells located in the zona facicolata of adrenal cortex to release the glucocorticoid into the blood (Smith and Vale, 2006). It is possible that the electro foot shock stress used in our study also induced such a mechanism. However, when the NAc shell was inactivated, there was an observable change in our results. Inactivation of the right side of the NAc shell inhibited stress effects, whereas inactivation of the left side exacerbated the stress effects (Figure 5). Neuronal pathway finding studies have not identified direct pathways between the NAc shell and hypothalamus paraventricular nucleus; however, one explanation for our findings is that the NAc shell is directly connected to the central nucleus of the amygdala (Koob 1999).

Investigators believe that these two parts of the brain, together with several forebrain areas including the basal nucleus of the stria terminalis, cooperate with each other as the extended amygdala, which is postulated to play a key role in the response of the brain to stress (Koob 1999). Considering this theory, we could hypothesize that inactivation of the NAc shell devastates the extended amygdala circuit and reduces the response to stress. These findings may indicate the laterality of the NAc shell in response to stress; indeed, in our previous study, we also found a kind of laterality within the NAc shell in response to morphine place conditioning in rats (Esmaeili et al., 2012). Other investigators have shown that the NAc is not a homogenous media in regards to dopamine, enkephalin, dynorphin, and substance P receptors (Voorn et al., 1994). This heterogeneity can also be functionally related to the different responses observed in our study. Our findings have functional importance because they provide a better understanding of the brain circuits that function in stress responses.

Present results also indicated that stress inhibits rats’ weight gain and reduces their food intake. These results agree with those of previous studies involving electro foot shock stress in male mice (Halataei et al., 2011) and rats (Hooshmandi et al., 2011). Investigators believed that the CRF released from the hypothalamic paraventricular nucleus during stress is the main neurohormone that mediates stress-induced anorexia (Kovács, 2013). Chronic stress in rodents may reduce food intake by such a mechanism and reduce weight gain as a result (Smith and Vale, 2006). In the present study, transient inactivation of the NAc shell inhibited the stress effect on weight gain but not on food intake. Both the left and right sides of the NAc shell responded similarly, with no indication of side bias. These results can be attributed to the involvement of the NAc shell in feeding behavior (Bello and Hajnal, 2010). Specifically, the transient inactivation of the NAc may have interacted with its role in feeding behavior; thus, one can conclude that the animals did not feed adequately because of NAc inactivity. These findings suggest that the NAc shell is not be an ideal site for investigations into the effects of stress on feeding. However, further research may help us to build a clearer picture of the NAc shell’s role in stress-induced effects on food intake.

Results obtained in the present study also indicated that stress increased the water intake in rats. This result is in agreement with those of previous studies (Sarahian et al., 2015). It is postulated that the vasopressin hormone released during stress from magnocellular cells located in the paraventricular nucleus is responsible for excessive water intake (Zimmermann et al., 2004). In our experiments, transient inactivation of the right side of the NAc shell inhibited the stress effect on water intake, whereas transient inactivation of the left side exacerbated the effect. This result is consistent with the results for corticosterone and indicated that transient NAc inactivation may inhibit or exacerbate the release of the vasopressin hormone. We did not measure the vasopressin hormone concentration in the animals because of the difficulty in reaching the hypothalamo-pituitary blood stream, but we suggest that this hormone should be measured in future experiments.

At last, the data indicated that stress reduces both locomotion activity and rearing in rats. Stress can induce freezing behavior in animals and amygdala is the main brain structure involved in this phenomenon (Roozendaal et al., 1996). Stress may directly interact with brain reward areas and inactivate the enzyme tyrosine hydroxylase (Czyrak et al., 2003), which indirectly inhibits D1 dopamine receptors that, when activated, increase dopamine-related behaviors such as locomotion, rearing, and sniffing (Czyrak et al., 2003). Based on these findings, it is acceptable to evaluate some of these activities as part of an indirect profile for stress effects (Czyrak et al., 2003; Mahdavi et al., 2014). However, in our study, transient inactivation of the NAc shell partly inhibited the stress effects on both locomotion activity and rearing, and the left side of the nucleus was more effective in this regard than the right. The shell of the NAc is believed to be involved in dopamine-related behaviors; thus, we can postulate that transient inactivation of this nucleus interferes with such behaviors. However, it must be noted that transient inactivation of the NAc shell did not affect these behaviors (Esmaeili et al., 2012).

In conclusion, this investigation indicated that the NAc shell interacts with stress effects on metabolic, hormonal, and behavioral responses, and we observed a clear laterality in the activity of the nucleus. According to these results, it is suggest that these findings should be considered in any future studies regarding stress and the NAc, particularly the NAc shell.
